# Covid-19: crisis, emotional governance and populist fantasy narratives

**DOI:** 10.1177/00471178221149634

**Published:** 2023-01-20

**Authors:** Catarina Kinnvall

**Affiliations:** Lund University

**Keywords:** anxiety, Covid-19, fantasy, populism, ontological insecurity

## Abstract

This short article discusses how different fantasy narratives have come together during the Covid-19 crisis in various far-right movements, parties and audiences across the world and how much of these fantasies rely on racialised and gendered notions of a fantastical world-order in which particular forms of emotional governance provide a relief and sense of security to certain societal groups. This involves a close engagement with crisis and crisis narratives in relation to ontological insecurity and anxiety; how such crisis narratives have materialised in fantasies related to borders and corona nationalism, and the emotional governance of these particular fantasies in the hands of populist leaders and their increasingly receptive audiences.

## Introduction

As some countries are slowly adapting to what looks to be a post-pandemic experience, a number of questions arise in terms of what this experience will entail for the future. How has the widely dispersed state of uncertainty changed underlying perceptions of self and others; what will a ‘new normality’ look like in the face of complex borders, anti-vaxx movements, and an increase in anti-liberal tendencies? To what extent has populist and authoritarian movements capitalised on the pandemic crisis narratives and what, if any, creative possibilities have this experience given rise to? These are some of the questions addressed in this short paper, which is focused on understanding the linkage between crisis, fantasy narratives and emotional governance with a particular emphasis on far-right populist movements and political leaders. That the pandemic has created a widely dispersed state of uncertainty is hard to refute. Coping with sickness and deaths, with quarantine, daily restrictions, face masks, border closure and distant emotional relations have challenged the everyday routines of individual citizens and have, for many, created a deep sense of insecurity; of not knowing what tomorrow holds – what Giddens^1^ refers to as ontological insecurity. However, behind these immediate effects of the pandemic experience lie less visible crisis scenarios narrated to the public as past and future happenings. Crisis narratives rely on imaginations, predictions and visions concerning what that future may look like, how particular pasts are to be blamed or glorified, and the specific actors responsible for the crisis. These fantasy narratives tend to converge in much far-right populist conceptions in which specific emotions become tied to fantastical visions of the past, present and future, involving everything from fabricated lies to myths and re-imagined memories of a past moral order. In this short paper, I discuss how such fantasy narratives have come together during the Covid-19 crisis in various far-right movements, parties and audiences across the world. The extent to which such fantasies rely on racialised and gendered notions of a fantastical world-order is here important for understanding how emotional governance can provide a relief and sense of security for certain societal groups. Hence, I discuss what we mean with crisis and crisis narratives in relation to ontological insecurity and anxiety; how such crisis narratives have materialised in fantasies related to borders and corona nationalism, and the emotional governance of these particular fantasies in the hands of populist leaders and their increasingly receptive audiences.

## Crisis narratives: Ontological insecurity and anxiety

The absence of political visions and the presence of unclear futures provide a foundation for crisis narratives to emerge and take hold. Crisis narratives can be both backward- and forward-looking. The forward-looking dimension often involves a vision of a forthcoming dystopia or catastrophe, but can also enable action and productive power, while the backward-looking dimension tends to envisage some elements of going back to an imagined uninterrupted past or at least to a previously defined present in terms of an imaginary status quo – a ‘foregoing normal’. However, a crisis can also produce a sentiment of existing in a void – being stuck in the ‘fixity’ of a calamity – a state that is often exacerbated by society’s initial emotional outburst in relation to a crisis, followed by a demand to return to normality.^2^ A crisis, in this sense, is both a concept and an event and has profound uncertainty as its underlying dimension. Global crisis narratives thus span across local, national and global contexts and can be of a more perceptible, such as wars, conflict, migration, the environment, a pandemic or of a more abstract kind, for example, economic recession, failing democracies, permanent poverty. As Chernobrov^3^ has argued, uncertainty itself can produce a crisis in the ontological narrative or it can be imagined in ways that reaffirm the crisis narrative. Feeling uncertain, at loss, or experiencing one’s existence as a void is likely to generate anxiety and frustration, an ontological insecurity focused on relief and redemption in order to escape falling into the ‘abyss’.^4^ However, such emotions do not occur in a vacuum. In relation to populist discourse, we can, with Moffitt,^5^ see ‘crisis as a phenomenon that is mediated and performed, and experienced culturally and socially’. This, what Moffitt^6^ calls the ‘spectacularization of failure’, entails for populist leaders and movements a separation of ‘“the people” from those who are responsible for the crisis’ and thus ‘legitimate their own strong leadership as a way to stave off or bring about an end to the crisis’. Crisis is thus not to be viewed as an exogenous event, an ‘outside shock’, which actors react to but as an endogenous construction ‘where ontological questions about the relationship between agent and structure are integrated and thus problematized’.^7^ As Resende^8^ argues in relation to the pandemic, this ‘spectacularization of failure’ has been actively created by populist forces in their ‘questioning of science, institutions and experts in relation to the virus’.

This is akin to John Cash’s^9^ description of how psychic defense mechanisms work in order to handle anxiety at the societal level through a process of unconscious social collusion and bargaining, where eventually a legitimate social defense mechanism is produced. Not only does such culturally mandated defense mechanisms become integral aspects of institutions, including the state, but they also work as an important way in which to control anxiety. They become, in other words, an aspect of intersubjective reality that exercises power over subjectivity.^10^ These defense mechanisms ‘kick in’ in response to an ontological insecurity set on constructing a secure image of what tomorrow holds, and can make masses and elites that may otherwise have little in common unite around an ideology that relieves them from the void by providing an apocalyptic and redemptive narrative vision of possible solutions to the crisis.^11^ Understanding such unconscious defense mechanisms requires an understanding of ontological security as a ‘security of becoming’ rather than a ‘security of being’ as originally understood by Giddens,^12^ in which the ontological insecurities people experience at times of crisis compel a ‘leap in faith’^13^ in terms of an imagined secure past and future that can relieve the individual from their present predicament.

Constructing such a ‘leap in faith’ has been evident in the crisis narratives of the populist right as detailed in a recent book by Bobba and Hubé^14^ covering eight European countries affected by the pandemic. Here the authors argue that in comparison to other crises, the pandemic did not lend itself to the traditional appeal to ‘the people’ as a basis for support. Instead, far-right populist leaders found themselves identifying new lines of conflict built on nationalism, and ‘neo-natalism’ with an emphasis on ‘we, the national people’ against both the EU and other member states. However, rather than confronting the pandemic per se, the far-right populist focus has often been on keeping the crisis narrative going in a continuous process of ‘naming, blaming, and claiming of systemic contradictions’ in a quest for crisis ownership.^15^ Owning the crisis thus requires a constant shift in terms of imagined solutions to the pandemic, in which fantasised objects of blame are to cover up for the inability to handle the insecurities at heart of the crisis.

## From crisis to fantasies: Border anxiety and corona nationalism

The term ‘fantasy’ can be understood in psychoanalytical terms as ‘a deep template in the unconscious mind of the individual which predisposes that person to experience people, events and situations in a particular way’.^16^ This means that fantasy is more than an escape from reality; rather it is ‘a constant and unavoidable component of real experiences’.^17^ The Covid-crisis is indeed a very real experience with more than 660 million being infected to date and more than 6.7 million deaths,^18^ and signifies a number of sub-structural crises, such as the lack of equipment and hospitals, lack of affordable health care and insurance, and exacerbated conditions of precarity for the most vulnerable worldwide. However, this ‘realness’ does not make it separable from fantasies about the crisis. Rather crises, like traumas, require significant psychological excertion for subjects to integrate traumatic events into an accessible interpretative framework and is often a step towards closure – a closing off of potential ambiguities and complexities of the social world.^19^ This is where fantasies come in as necessary but always imagined responses to a crisis narrative in order to bring uniformity and significance to our existence.^20^ As Allen^21^ has argued, such essential fantasies can be more or less certain about the closures they provide as well as about the ‘realness’ of the reality they bring into being. As fantasies they are tied to the institutionalisation of shared collective identities, or master signifiers, and stimulate members to experience shared emotions and united understandings of a situation or event.^22^ The ways in which particular fantasies come to dominate is thus related to their emotional and structural history. In this sense anxiety can be ‘healed’, and a sense of ontological security restored, through fantasies of closure that string an emotional and structural chord with their subjects. Nations and national borders constitute particular powerful fantasies in their attempts to objectify the state, and state borders, by imagining it as a person or a group writ large.

The pandemic has thus been yet another instance in which fantasies of secure borders – borders as being ink on the map, rather than lines in the sand – become imaginary anxiety-reducing responses. This ‘corona-nationalism’ has been geared towards those perceived to be outside of these borders, so called ‘fantasmatical’ others^23^ – often in relation to ascribed national fantasies. Such border anxieties are clearly visible in terms of how contagious diseases have been ‘othered’: the Spanish flu, the Ebola as ‘African’, the coronavirus as ‘Chinese’. In terms of the Covid-19 pandemic, those on the other side of the border (any border) have been perceived as a threat, as carriers of Covid-19, while the border itself is presented as a protective tool – an anxiety-reducing mechanism. However, the spread of the virus has also affected internal othering, as witnessed in the blaming of Muslim minorities in India – defined as ‘super-carriers’– after the Muslim religious organisation, the Tabligh-et-Jamaat, had its main gathering in March 2020. This can be compared to other election meetings and religious gatherings in India, especially the 2021 Kumbh Mela, supported by the current far right Hindu nationalist government in which the majority is perceived as innocent, or at least neutral, in the national crisis narrative. Similarly, in the US under Trump, Latinos were characterised as the vectors of the disease at the same time as more than 1700 anti-Asian hate incidents were reported, while in Europe the Roma, but also migrants more generally, were blamed for spreading the virus.^24^

Noticeable during the Covid-19 pandemic is how fantasies of external others spread through the circulation of memes and social media commentary, scapegoating entire populations as being responsible for the virus and its spread. In more specific terms, and related to far-right populism, is how fantasies of clear objects were imagined as being either reclaimable or restorable in order to establish a cohesive sense of identity and community.^25^ In addition to a process of ‘naming’ abject others, this has also involved a persistent ‘shifting of blame’ and a continuous promise to ‘victim-perpetrator reversal’.^26^ In terms of the pandemic, it has exposed dehumanising fantasies about ‘dirty’ immigrants carrying diseases,^27^ while the rapid spread of the virus among local inhabitants often has remained unproblematised.

## From populist fantasies to the emotional governance of Covid-19

In protests around the world against anti-lockdown measures, we have seen how anti-vax, conspiracy theories and QAnon have merged together in a number of fantasy narratives and how these have gained followers across contexts. QAnon, together with other apocalyptic conspiracy movements, rely on the crowdsourcing of crisis narratives. This is what led the World Health Organisation to describe the shift from a pandemic to an ‘infodemic’ – the spreading of false information, or fantasies, about Covid-19.^28^ These fantasies ranged from Covid-19 being a ‘Chinese bioweapon’; a weapon ‘planned by the Bill and Melinda Foundation’; a ‘vaccine experiment’; a ‘media-constructed virus’, to evangelical claims that ‘only those not chosen by God would be affected by the disease’.^29^ Underlying such claims and uniting the protesters worldwide, seemed to be a circulation of collective experiences of anger and resentment. Experiences directed not just at the establishment but also at particular others, such as immigrants, Muslims, Latinos, Chinese. This suggests that ‘emotions are not simply “within” or “without” but that they create the very effect of the surfaces or boundaries of bodies and worlds’.^30^ Fantasies thus supply a particular form of emotional governance in the far-right populist narrative by delineating ‘the bodies of individual subjects and the body of the nation’^31^ This, what Richards^32^ refers to as an ‘emotional public sphere’, specifies the detailed emotions involved in these delineations, such as ‘fear, hatred, and contempt towards outsiders’.^33^

In terms of far-right, and in most cases anti-elite populist discourses, emotional governance takes a number of forms. One is the organisation of unease, fear and resentment, which under the pandemic has come to include areas related to health and social protection. In the case of Italy for instance, the far-right party, the Lega, changed the fantasies of the unknown, different and foreign in the public sentiments to fathom a domestic fear – the fear of the virus and by extension the fear of every person. The message conveyed of the Lega was ‘that any recovery from the economic distress caused by the pandemic needs to go through more security, more discipline, “more unkindness”, “zero tolerance” for criminals, and particularly for foreigners and threats to national borders’.^34^ Another significant dimension of emotional governance and far-right populist discourse during the pandemic has been connected to the fantasies involved in non-mainstream information. In country after country, we have witnessed how presentations of different versions of scientific expertise were deployed and communicated in response to the crisis. Accusing establishments of ‘fake news’ deployed to manipulate the electorate or as governmental conspiracies to keep citizens unaware of ‘real’ treatments to avoid catching Covid-19, has meant portraying them as being behind the misery. Continuing with the Italian example, the leader of the Lega, Matteo Salvini, thus ‘repeatedly shared alleged success stories and updates on the plasma treatment for Covid-19’, ‘opposing the “arrogance” and “inadequacy” of “intellectuals” and “schoolmarms” in the government’.^35^ Both features contributed to the projection and dissemination of an ‘impending doom’ that if crossed over ‘cannot be reversed’.^36^ This is what make far-right portrayals of political community as ultimately grounded in national belonging unique and an ultimate ‘fantasmatical’ resource of ontological security.

As Sara Ahmed^37^ asserts in her description of affective economies, ‘emotions *do things*, and they align individuals with communities—or bodily space with social space—through the very intensity of their attachments’. Attaching a sign to a body, by constituting it as an object of fear, is part of a racialised and gendered fantasy of ‘the ordinary’ (whiteness, masculinity, traditional values, etc.) as being under threat. In the far-right populist fantasy, it is such ordinary persons that constitute ‘the real victims’. ‘The white subjects claim the place of hosts (“our shores”) at the same time as they claim the position of the victim, as the ones who are damaged by an “unmerciful government”’.^38^ This is a process in which ideological and cultural fantasies are internalised and increasingly normalised. However, this does not automatically imply that far-right populist forces have been ultimately successful in taking ownership of the pandemic crisis. As Bobba and Hubé’s^39^ eight country study of populism and Covid-19 reveals, the pandemic crisis has also provided space for forms of political collaboration or ‘non-hostile’ tacit agreements in the name of national and international solidarity. While this may provide some temporary comfort, it does not fundamentally challenge an increasingly normalised political reality of closure, unity and homogeneity as the ultimate narrative resort to exclusionary politics.

## Notes

1. Anthony Giddens, *Modernity and Self-Identity* (Cambridge: Polity Press, 1991).

2. Annika Bergman Rosamond, Thomas Gammeltoft-Hansen, Mo Hamza, et al., ‘The Case for Interdisciplinary Crisis Studies’, *Global Discourse*, 12(3–4), 2022, pp. 465–486. Yael Danieli, Danny Brom and Joe Sills, ‘The Trauma of Terrorism’, *Journal of Aggression, Maltreatment & Trauma*, 9(1–2), 2004, pp. 1–17. Jenny Edkins, ‘Forget Trauma? Responses to September 11’, *International Relations*, 16(2), 2002, pp. 243–56.

3. Dmitry Chernobrov, ‘Ontological Security and Public (Mis)Recognition of International Crises: Uncertainty, Political Imagining, and the Self’, *Political Psychology*, 37(5), 2016, pp. 581–96.

4. Giddens, *Modernity and Self-Identity*.

5. Benjamin Moffitt, ‘How to Perform Crisis: A Model for Understanding the Key Role of Crisis in Contemporary Populism’, *Government and Opposition*, 50(2), 2015, pp. 189–217, p. 195.

6. Moffitt, ‘How to Perform Crisis’, p. 198.

7. Erica Simone Almeida Resende, ‘Pandemics as Crisis Performance: How Populists Tried to Take Ownership of the COVID-19 Pandemic’, *Mezinárodní vztahy. Czech Journal of International Relations*, 56(4), 2021, pp. 147–56, p. 151.

8. Resende, ‘Pandemics as Crisis Performance’.

9. John Cash, ‘Psychoanalysis, Cultures of Anarchy, and Ontological Insecurity’, *International Theory*, 12(2), 2020, pp. 306–21.

10. Cash, ‘Psychoanalysis, Cultures of Anarchy, and Ontological Insecurity’.

11. Elisabeth Young-Bruehl, *The Anatomy of Prejudices* (Cambridge, MA: Harvard University Press, 1996).

12. Giddens, *Modernity and Self-Identity*. This distinction is further developed in Catarina Kinnvall, ‘Ontological Insecurities and Postcolonial Imaginaries: The Emotional Appeal of Populism’, *Humanity and Society*, 42(4), 2018, pp. 1–21.

13. Badredine Arfi, ‘Existential Security *qua* Surviving (While Always Becoming Otherwise) Through Performative Leaps of Faith’, *International Theory*, 12(2), 2020, pp. 291–305.

14. Giuliano Bobba and Nicolas Hubé, *Populism and the Politicization of the COVID-19 Crisis in Europe* (Cham, Switzerland: Springer, 2021).

15. Bobba and Hubé, *Populism and the Politicization of the COVID-19 Crisis in Europe*.

16. Barry Richards, ‘Psychological Underpinnings of Post-Truth Politics’, *International Journal of Media & Cultural Politics*, 14(3), 2018, pp. 401–6, p. 402.

17. Amy Allen, *Critique on the Couch: Why Critical Theory Needs Psychoanalysis* (New York, NY: Columbia University Press, 2020). See also Catarina Kinnvall and Ted Svensson, ‘Exploring the Populist ‘Mind’: Anxiety, Fantasy, and Everyday Populism’, *The British Journal of Politics and International Relations*, 24(3), 2022, pp. 1–17. DOI: 10.1177/13691481221075925.

18. Worldometer, Corona Virus Update, 8 January 2023, available at: https://www.worldometers.info/coronavirus/ (accessed 8 January 2023).

19. Jakub Eberle, ‘Narrative, Desire, Ontological Security, Transgression: Fantasy as a Factor in International Politics’, *Journal of International Relations and Development*, 22(1), 2019, pp. 243–268.

20. Mari Ruti, *A World of Fragile Things: Psychoanalysis and the Art of Living* (Albany, NY: State University of New York Press, 2009).

21. Allen, *Critique on the Couch*.

22. Reinhart Wolf, ‘Identifying Emotional Reactions to Status Deprivations in Discourse’, *International Studies Review*, 19(3), 2017, pp. 491–6. For an extended discussion of fantasy and emotional governance, see Kinnvall and Svensson, ‘Exploring the Populist ‘Mind’’; Catarina Kinnvall and Ted Svensson ‘Trauma, Home and Geopolitical Bordering: A Lacanian Approach to the Covid-19 Crisis’, *International Studies Quarterly*, forthcoming 2023, and; Pasko Kisic Merino and Catarina Kinnvall, ‘Governing Emotions: Hybrid Media, Ontological Insecurity and the Normalisation of Far-Right Fantasies’, *Alternatives*, 2022, online first, https://doi.org/10.1177/03043754221123467.

23. Jacques Lacan, *The Four Fundamental Concepts of Psychoanalysis* (London: The Hogarth Press, 1977).

24. Claire Thomas, ‘COVID-19 Emergency Relief Must Reach Everyone, Including Minorities and Indigenous Peoples’, *Minority Rights Group International*, 24 March 2020, available at: https://minorityrights.org/2020/03/24/covid19-leave-no-one-behind/ (accessed 25 March 2022).

25. Eberle, ‘Narrative, Desire and Ontological Security’, p. 255.

26. Ruth Wodak, *The Politics of Fear: The Shameless Normalization of Far-Right Discourse* (London: SAGE, 2021), p. 95.

27. Taylor Woods, Robert Schertzer, Liah Greenfeld, et al., ‘COVID-19, Nationalism, and the Politics of Crisis: A Scholarly Exchange’, *Nations and Nationalism*, 26(4), 2020, pp. 807–25.

28. Marc-André Argentino, ‘QAnon Conspiracy Theories About the Coronavirus Pandemic are a Public Health Threat’, *The Conversation*, 8 April 2020.

29. Argentino, ‘QAnon Conspiracy Theories’. See also Richard Orange, ‘Why Sweden had an Anti-Lockdown Protest Without Having a Lockdown’, *The Local*, 8 March, available at: https://www.thelocal.se/20210308/explainer-why-sweden-had-an-anti-lockdown-protest-without-having-a-lockdown/ (accessed 29 May 2021).

30. Sara Ahmed, *The Cultural Politics of Emotion* (London: Routledge, 2014), pp. 117–8.

31. Ahmed, *The Cultural Politics of Emotion*.

32. Barry Richards, ‘Extreme Nationalism and the Hatred of the Liberal State’, in Nicolas Demertzis (ed.), *Emotions in Politics: The Affect Dimension in Political Tension* (London: Palgrave, 2013), pp. 124–42, p. 126.

33. Neta C. Crawford, ‘Institutionalizing Passion in World Politics: Fear and Empathy’, *International Theory*, 6(3), 2014, pp. 535–57, p. 536.

34. Giacomo Bottos, Eleonora Desiata and Andrea Pareschi, ‘The Re-Shaping of the Political Discourse in Times of Crises: The Italian laboratory’, *FEPS Covid Response Papers*, October 2020/ #9, p. 10, available at: https://www.feps-europe.eu/attachments/publications/political%20discourse_feps%20covid%20response%209.pdf (accessed 8 May 2021).

35. Bottos et al., ‘The Re-Shaping of the Political Discourse in Times of Crises’, p. 11.

36. Benjamin Moffitt, *The Global Rise of Populism: Performance, Political Style, and Representation* (Stanford, CA: Stanford University Press, 2016).

37. Sara Ahmed, *The Cultural Politics of Emotion* (London: Routledge, 2014), p. 119.

38. Ahmed, *The Cultural Politics of Emotion*, p. 118.

39. Bobba and Hubé, *Populism and the Politicization of the COVID-19 Crisis in Europe*.

